# Bioturbation by the Ghost Shrimp *Lepidophthalmus louisianensis* Increases Petroleum Hydrocarbon Degradation for Coastal Sediments in Mildly Oiled Mesocosms

**DOI:** 10.3390/microorganisms14030695

**Published:** 2026-03-19

**Authors:** Nihar R. Deb Adhikary, Paul L. Klerks, Andrei Y. Chistoserdov

**Affiliations:** Department of Biology, University of Louisiana at Lafayette, P.O. Box 43602, Lafayette, LA 70504-3602, USA

**Keywords:** polycyclic aromatic hydrocarbon, bioturbation, naphthalene degradation, 16S rRNA gene, *Rhodobacteraceae*

## Abstract

Bioturbating animals move around large amounts of sediment, changing its physicochemical properties and biogeochemical processes. The present study assessed the role of the ghost shrimp *Lepidophthalmus louisianensis*, a major coastal bioturbator in the Northern Gulf of Mexico, in the fate of crude oil after the 2010 Deepwater Horizon blowout. Experiments were conducted in greenhouse mesocosms, with or without ghost shrimp and with or without added oil, reflecting mild surface or subsurface oiling in a beach environment. To evaluate the hydrocarbon-degradation potential of the sediment microbial community, a respirometric radiotracer assay was conducted with ^14^C naphthalene as a model polycyclic aromatic hydrocarbon (PAH) compound. Oil augmentation led to a substantial increase in the PAH degradation potential of mesocosm sediments, which was further enhanced by the presence of the bioturbator. However, bioturbation alone, without previous oil exposure, did not enhance naphthalene degradation. 16S rRNA gene analyses showed that there were no significant changes in the microbial community composition associated with either bioturbation, oil augmentation, or both. This study demonstrated bioturbation- and oil-exposure-related enhancement in hydrocarbon degradation in mildly oiled sediment, and indicated that this may be due to an increased expression of PAH degrading activities in the preexisting community of hydrocarbon-degrading bacteria rather than resulting from a shift in the microbial community composition.

## 1. Introduction

The Deepwater Horizon (DWH) oil spill in the northern Gulf of Mexico (GoM) was the largest oil spill in the history of the petroleum industry, with approximately 750 million liters of oil released in the northern GoM in 2010 [1]. Despite intensive initial clean-up [2], most of the oil remained in the system and became subject to microbial degradation, either in the water column or on the seafloor. A large amount of oil has reached the coastline and coastal salt marshes [3,4].

Various environmental factors influence oil biodegradation, including the presence of nutrients and oxygen, and physical factors like wind and wave action. Microorganisms catabolize oil components using both aerobic and anaerobic pathways. The aerobic processes are much faster and well-studied [5,6], but anaerobic degradation may also be important in the deep anoxic sediments [7]. While microbial degradation is clearly dependent on various physical and chemical characteristics of the sediment, benthic organisms can potentially affect both the sediment characteristics and the microbial community in sediment matrices. Coastal benthic macrofauna overturn sediment as they construct and maintain burrows or process sediment while feeding. They also increase the exchange of water between the sediment and the water column, which in turn increases oxygen penetration into the sediment [8]. Temperature gradients and the biogeochemical cycling of nutrients are also affected by bioturbation. Consequently, bioturbating organisms have a direct impact on the microbial community [9,10,11].

The fact that sediment bioturbators can change local microbial populations has been demonstrated for the polychaete *Nereis diversicolor* [12,13,14]. Moreover, several studies have indicated that polychaete bioturbation increases both the abundance of hydrocarbonoclastic bacteria (also known as hydrocarbon-degrading bacteria, e.g., [15]) and the rate of oil removal [13,16,17,18,19,20]. Other bioturbators, including crustaceans and bivalves, also appear to alter microbial community composition and abundance [10,12,15,21,22,23,24]. However, their role in oil degradation is still not clear; one study indicated that microbial community changes increased oil loss, while others did not detect such an enhancement of oil degradation [14].

Bioturbators are very abundant in the intertidal and subtidal zones of the GoM, and, therefore, may affect the fate of oil introduced by the DWH disaster. The ghost shrimp *Lepidophthalmus louisianensis* is one of the most important bioturbators along the northern GoM coast. This species is widely distributed here, often present at very high densities (up to 33 ind./m^2^), makes deep burrows (up to 3 m), and is a very active bioturbator with sediment turnover rates up to 100 kg dry sediment/m^2^/year [25,26]. After the DWH blowout, a portion of the oil was deposited on parts of the northern GoM shoreline, while oil was also deposited on subtidal sediment [3]. The high abundance of ghost shrimp in these areas prompted the present study on the role of bioturbation with respect to oil biodegradation. We hypothesized that ghost shrimp enhances PAH degradation via bioturbation-induced microbial activity, with or without altering community composition. The study was conducted using laboratory mesocosms and focused on PAHs, as these hydrocarbons are among the most toxic and recalcitrant components of the oil. Specific objectives were to (i) assess the influence of ghost shrimp on the degradation of PAHs, using ^14^C naphthalene in a respirometer assay [27]; and (ii) determine the changes in microbial community composition using second-generation sequencing of the bacterial 16S rRNA genes in order to elucidate a possible diversity shift and enrichment of potential oil degraders induced by the oil and by the bioturbator.

## 2. Methods

### 2.1. Sampling Site

Sediment, seawater, and ghost shrimp for the greenhouse mesocosms were collected from Bay St. Louis, MS, USA (30.25658° N, 89.41510° W), over multiple collection trips. Water temperature ranged from 14.0 to 31.1 °C; salinity ranged from 0.9 to 26.4 ppt. Collected animals were brought to the lab in their ambient seawater. In the lab, water temperature and salinity were gradually adjusted to the experimental conditions (see below).

### 2.2. Mesocosm Setup

Greenhouse mesocosms were set up in a greenhouse at the Lafayette Ecology Center (University of Louisiana at Lafayette). The mesocosms used 114L all-glass tanks (PetSmart, Idaho Falls, ID, USA) (60 cm L × 30 cm W × 60 cm H), with sediment and seawater from the collection site. The salinity in the mesocosms was gradually adjusted (a change of up to 2 ppt per day) to about 15 ppt, and maintained at 14.5–15.5 ppt throughout the study. Slow aeration was provided in all tanks. Experiments were conducted using a total of 16 mesocosms and the following four treatments: (a) ghost shrimp present, oil added (5 replicates); (b) ghost shrimp present, no oil added (5 replicates); (c) no ghost shrimp, oil added (3 replicates); and (d) no ghost shrimp present, no oil added (3 replicates). Two different types of mesocosm experiments were conducted for the present study. The first type of experiment used oil at the sediment surface, reflecting the scenario where oil washes ashore in an intertidal area inhabited by ghost shrimp. The second type of experiment had oil as a subsurface layer in the sediment, reflecting the scenario where oil has penetrated the sediment. Both surface oil and subsurface oil were previously reported for northern GoM coastal areas [3]. For the surface-oil experiments, mesocosms were filled with sediment (35 cm depth) and 20 cm of overlying seawater and allowed to equilibrate for 2–5 weeks prior to the addition of ghost shrimp. Eight adult ghost shrimp were then added per mesocosm for the two ghost-shrimp-present treatments. Animals that did not burrow within a day were considered to be unhealthy and replaced with new animals. These animals were left to acclimate for 7 or 14 days before oil was added. Mesocosms were dosed with Marlin Platform Dorado oil, a surrogate for the Macondo MC252 sweet crude oil released in the DWH incident. Prior to dosing, the oil was sparged with air for 48 h to simulate mild weathering. A total of 3 mL of oil was mixed with 300 g of dry sediment, and the resulting slurry was applied on top of the sediment surface of the mesocosms for the oil-added treatments. The PAH composition of the sparged oil and the dosing sediment were reported previously [28].

For the subsurface-oil experiment, the mesocosms were only partially filled with sediment up to 30 cm depth, and then water was added. The mesocosms in the ghost-shrimp treatments received 5 ghost shrimp, which were allowed to acclimate for a week. A total of 5 mL of oil mixed with 300 g of air-dried sediment was applied to the top of each mesocosm in the oil-added treatments. An additional layer of sediment (~15 cm) was carefully added without disturbing the oil layer. Three additional shrimp were then added to the ghost-shrimp treatments (so that these mesocosms also had a total of 8 ghost shrimp per mesocosm, with shrimp present both above and below the oil layer). The final concentrations of oil in the sediments ranged from 31 to 52 ng/g, as determined by GC analyses [29].

Once both the oil and ghost shrimp were present, exposure lasted 10–15 days depending on time of year and resulting temperature (as oil degradation is expected to be inversely related to temperature). The two surface-oil experiments lasted 10 and 15 days (during these experiments, mesocosm temperatures averaged, respectively, 28.3 and 18.2 °C), while the subsurface-oil experiment lasted 11 days (with water temperature averaging 27.4 °C). Temperature and salinity were monitored daily.

### 2.3. Sediment Sampling from Mesocosms for Radiotracer Assay

For the radiotracer assay, sediment was collected at the end of each experiment. Sediment samples were collected from randomly chosen areas in a tank using a sediment core, as described below, and consisted of a combination of surface and subsurface sediment. Each sample was divided into triplicate subsamples to set up radiotracer incubation.

### 2.4. Respirometric Assay Design

These assays used a modified version of the respirometric assay used by Reid et al. [27]. Wide-mouth glass jars (110 mL capacity) with air-tight screw caps (containing a septum) were used as respirometers. The assay quantified ∑^14^CO_2_ produced from ^14^C-naphthalene mineralization by the microbes present in the sediment at an ambient temperature of 25 °C. Since these were static (non-flow-through) respirometers, separate jars were used for each time point (0 h and 8 h). In preliminary experiments, linear changes in ∑^14^CO_2_ emissions were observed within the first 12 h, after which the response plateaued, probably due to oxygen limitations. Therefore, an incubation time of 8 h was chosen. For each sediment sample and each time point, three replicates were used. Twenty grams of wet sediment was placed in a glass jar along with a 20 mL glass scintillation vial with 5 mL of 1 M NaOH to trap ∑^14^CO_2_ (forming Na_2_^14^CO_3_), and the jar lids were tightened. Each sample was spiked with 0.045 µCi of ^14^C naphthalene (ARC 1260 Naphthalene [1-^14^C], American Radiolabeled Chemicals, St. Louis, MO, USA) through the lid septum using a microsyringe. The final concentration of the radiotracer in the sediment was 0.79 pmol, which was not expected to change the biogeochemical processes in the sediment. Microbial reactions were stopped by injecting 2 mL of 2 M phosphoric acid into the sediment at 0 h (i.e., immediately after adding the ^14^C naphthalene), and after 8 h of incubation. The glass jars were then shaken vigorously (but carefully to avoid spilling the NaOH in the trap), so that the residual ^14^CO_2_ was released and absorbed in the trap. Scintillation cocktail (5 mL of ScintiVerse^TM^ BD cocktail, Fisher Scientific, Waltham, MA, USA) was added to each vial. Quantities of trapped ^14^C were then measured in a scintillation counter (Beckman-Coulter, Brea, CA, USA) and corrected for quenching and counting efficiency. The radioactivity was converted to picomoles of naphthalene degraded per gram of sediment per day.

### 2.5. DNA Isolation

These analyses were done for both the surface-oil and subsurface-oil experiments. A small amount of sediment (~200 mg) was collected from every mesocosm for DNA isolation on day 10 only. Sediment samples were collected using sediment cores at two separate depths: the sediment surface (top 2 cm) and subsurface (at a depth of about 7 cm). Collected samples were brought to the lab on ice, and microbial DNA was extracted using a Powerlyzer^®^ Powersoil^®^ DNA Isolation Kit (Qiagen, Carol Stream, IL, USA), according to the manufacturer’s protocol. The extracted DNA quantities were measured with a NanoDrop^®^ ND-1000 spectrophotometer (ThermoFisher Scientific, Waltham, MA, USA) and then stored at −20 °C until further use. In total, we analyzed 77 samples from three independent microcosm experiments: 22 samples for the “shrimp +, oil −” treatment (GS); 25 samples for the “shrimp +, oil +” treatment (GS + O); 14 samples for the “shrimp −, oil +” treatment (O); and 16 samples for the “shrimp −, oil −” controls (NS + NO).

### 2.6. 16S RNA Sequencing

Isolated DNA was sent to the University of Michigan Medical School Host Microbiome Initiative for 16S rDNA library preparation and sequencing. DNA libraries were generated per Illumina’s protocol for the MiSeq platform. The 16S rRNA gene sequencing was done by amplifying the V4 region using dual-indexing technology [30]. The number of raw sequences in all libraries was uniform, with an average of 4000 ± 350 sequences per library.

### 2.7. Operational Taxonomic Unit Analysis

The fastq file, which was generated as output after 16S rRNA gene sequencing, was “cleaned”, “denoised”, and processed using the Mothur package v1.48.0 [31]. The sequences were aligned to the SILVA reference alignment, OTUs were generated, and OTUs were then taxonomically classified with 97% similarity using the GreenGenes database [32].

### 2.8. Statistical Analysis

All data visualization was done exclusively in R v4.1 using ggplot2 v4.0.2 and Vegan packages v2.7-3, except that naphthalene degradation rates were compared between treatments using one-way ANOVA in the R package and an ANOVA calculator (One-Way ANOVA Calculator and Tukey’s HSD; https://www.statskingdom.com/180Anova1way.html (accessed on 24 October 2025)). Prior to analysis, the assumptions of ANOVA were evaluated. Normality of the residuals was assessed using the Shapiro–Wilk test, which indicated a slight deviation from normality (*p* = 0.032). Homogeneity of variance among treatments was tested using Levene’s test, and the results showed that the assumption of equal variances was satisfied (*p* = 0.262). Although the residuals showed minor deviation from normality, ANOVA is generally robust to moderate violations of this assumption when variance homogeneity is met. Therefore, a one-way ANOVA was conducted to examine differences among treatments. The analysis revealed a significant effect of treatment on the measured variable (F = 118.67, *p* < 0.001), indicating that mean values differed significantly among the treatments. The ANOVA output file is shown in Table S1. Output from the Mothur platform was imported into Phyloseq for further OTU analyses [33]. Alpha diversity, observed OTUs, Chao1 [34], and Shannon [35] coefficients, were calculated for the microbial communities in all treatment types (“shrimp +, oil −”, “shrimp +, oil +”, “shrimp −, oil +”, and “shrimp −, oil −”). Using Mothur, Phylip-formatted thetaYC-based distance matrices were created for the processed 16S rRNA data to elucidate similarities among samples. The thetaYC distance matrix was then used to conduct principal coordinate analyses (PCoA) of the communities. The analysis of molecular variance (AMOVA) was used to find clustering treatment types [36].

## 3. Results and Discussion

The goal of this study was to ascertain whether bioturbation by ghost shrimp enhances PAH degradation in the northern GoM, and to understand the underlying microbial changes. Naphthalene was chosen as a model PAH compound because it degrades relatively fast in the sediment (see below), is available in a labeled form, and certain enzymes (the PAH ring-hydroxylating dioxygenases) that attack more complex PAHs also initiate naphthalene degradation [37,38]. Two different methods were used for oil application to the mesocosms designed to simulate (1) oil deposited from the water column by the tide (surface application), and (2) oil trapped in the sediment following a storm event (subsurface application).

### 3.1. Naphthalene Degradation in Bioturbated and Non-Bioturbated Sediments

**Figure 1:**
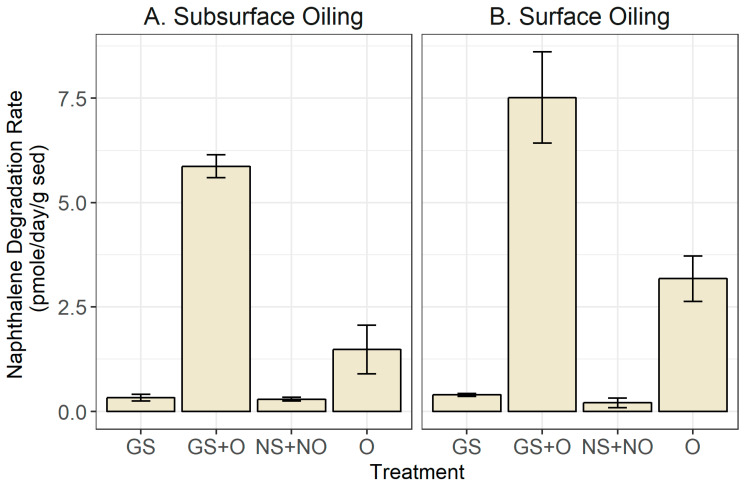
Naphthalene degradation rates (in picomoles per gram sediment per day—means ± S.E.), for sediment from mesocosms with or without ghost shrimp and with or without oil added. Oil was added to the sediment subsurface (**A**) or to the surface (**B**) of mesocosms with sediment and seawater. The sediment was collected 10–15 days after the addition of the oil. To measure naphthalene degradation, 20 g of sediment was spiked with labeled (^14^C) naphthalene in air-tight containers and naphthalene degradation was measured by the amount of ^14^CO_2_ produced over 8 h. Treatments: “GS”, “GS + O”, “NS + NO”, and “O”, representing “shrimp +, oil −”, “shrimp +, oil +”, “shrimp −, oil −”, and “shrimp −, oil +”, respectively. All analyses were performed in triplicate. One-way ANOVA-based *p* values for the “GS + O”, “O”, and control (“GS” or “NS + NO”) treatments were less than 0.001 (*p* < 0.001) in both experiments.

Regardless of whether oil was added to the sediment surface or deposited into the subsurface, bioturbation by ghost shrimp positively affected naphthalene degradation rates (Figure 1). For the experiment in which oil was added to the sediment surface, sediment from oiled mesocosms with ghost shrimp had a two-fold higher naphthalene degradation rate than that for sediment from the oiled mesocosms without ghost shrimp (*p* value < 0.001) (Figure 1). For sediment from mesocosms with just bioturbators (i.e., no oil present), the PAH degradation was very slow and comparable to that of the control mesocosms with neither shrimp nor oil. These results indicate that previous sediment exposure to oil is critical for the induction of PAH degradation; the naphthalene degradation rate increased 15-fold after crude oil addition to non-oiled sediments. For the experiment with the subsurface oil in the mesocosms, the results were qualitatively similar to those for the experiment with the surface oil. For the oiled mesocosms, the earlier presence of ghost shrimp in this sediment further stimulated naphthalene degradation (*p* value < 0.001) (Figure 1). Similarly, the presence of oil in the mesocosms resulted in an increased degradation rate compared to that for the unoiled control (Figure 1). A notable difference between the two experiments was a more prominent (three-fold) stimulation of naphthalene removal by the bioturbator in the case of the subsurface oil. One possible explanation for this phenomenon is that burrowing introduces oxygen into deeper sediments, thereby stimulating PAH oxidation. Thus, it appears that ghost shrimp may play an even more important role in removing subsurface oil than in the case of surface oil.

The present study’s findings are consistent with several previous studies showing that bioturbation by polychaetes enhances oil removal from sediments [16,18]. An in situ study on the lugworm *Arenicola marina* in sandy beaches confirmed this bioturbation-enhanced biodegradation and also showed that bioturbation evens out the distribution of oil in the sediment [39]. Its burial of surface oil to a depth of 10–20 cm stimulated both microbial and physical degradation of hydrocarbons [39]. PAH degradation potential has been shown to be greater for burrows of two species of polychaetes, a mollusk and an enteropneust, rather than that in non-burrowed sediment [16]. Polychaete studies have also been focused on other oil-component degradation (e.g., bulk oil). Studies with different bioturbating species have shown that feeding habits, burrowing strategy, burrow shape, burrow size, and burrow density all impact the fate of oil components [40]. Because the ghost shrimp used in this study makes deep burrows and its activity differs from that of polychaetes with respect to all those variables, it is risky to make precise predictions of the relative magnitude of their impact. Since *L. louisianensis* makes deep burrows (up to 3 m deep), actively ventilates these burrows, and turns over large amounts of sediment (up to 100 kg dry sediment/m^2^/year for a dense population; [26]), it is anticipated that its impact may exceed that of polychaetes in areas where ghost shrimp are common.

### 3.2. The Diversity and Changes in Bacterial Community Composition After Addition of Oil and Bioturbators

In the present study, both bioturbation and previous oil exposure increased naphthalene degradation rates. Such an increase can be due to changes in the microbial community composition or simply due to an induction of naphthalene-degrading ability in the existing microbial community. Metagenomic analyses of microbial communities in heavily oiled sediments detected an increase in key pathways involved in hydrocarbon metabolism [41,42]. We monitored the dynamics of the microbial community in the different mesocosms by second-generation 16S rRNA gene sequencing. There were, on average, over 60 major phyla present in each sample. Around 10% of the total community memberships in every sample remained unidentified after the OTUs were classified using the GreenGenes reference taxonomy tool.

**Figure 2:**
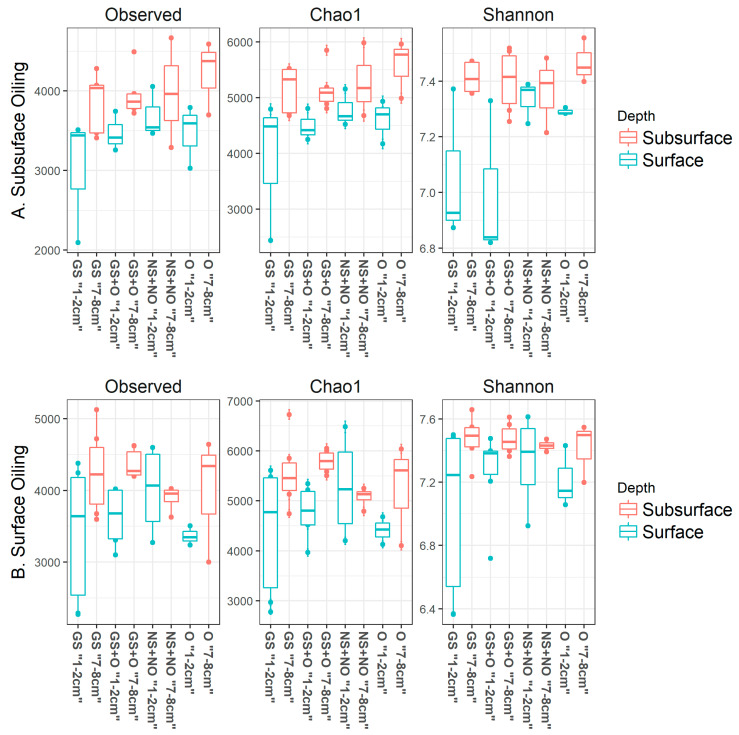
Alpha diversity metrics (observed number of OTUs, Chao1, and the Shannon diversity indices) are shown as boxplots. Data are for samples collected from two separate greenhouse mesocosm experiments with oil added to subsurface (**A**) or surface (**B**) of the mesocosms. Samples were collected from four treatments: “GS”, “GS + O”, “NS + NO”, and “O”, representing “shrimp +, oil −”, “shrimp +, oil +”, “shrimp −, oil −”, and “shrimp −, oil +”, respectively, at two depths of “1–2 cm” and “7–8 cm”.

**Figure 3:**
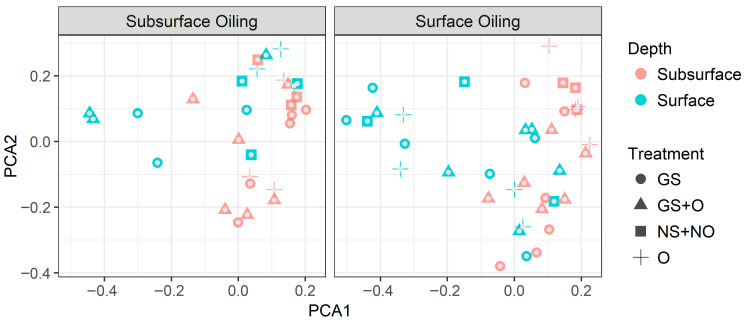
Two-dimensional principal coordinate plots based on thetaYC-based distance matrices, which compare microbial communities for day 10 (10 days after oil addition) libraries for subsurface- and surface-oiling experiments. Treatments: “GS”, “GS + O”, “NS + NO”, and “O”, representing “shrimp +, oil −”, “shrimp +, oil +”, “shrimp −, oil −”, and “shrimp −, oil +”, respectively. No specific patterns were observed.

Alpha diversity was greater in the samples collected from the 7–8 cm depth than in those from 1 to 2 cm (Figure 2A,B) in all treatment groups, and in both greenhouse mesocosm experiments with oil applied at two different depths (subsurface and surface). However, the difference in alpha diversity between sediment depths was greater when the oil was applied to the subsurface (Figure 2A). Nevertheless, a principal component analysis of microbial community based on a thetaYC-based distance matrix did not show any distinct difference between treatment groups or patterns attributed to individual communities (Figure 3). Consequently, neither the bioturbation nor the oil addition appeared to have caused any major shifts in the bacterial community structure.

Our results contrast with the finding that the polychaete *H. diversicolor* changes the structure of microbial communities in nature [13]; moreover, the same organism promotes the diversity of hydrocarbonoclastic bacteria [20]. Similar results have been observed in microcosm experiments with *H. diversicolor*, which demonstrated that this polychaete initiates a shift in microbial communities to a dominance of Gammaproteobacteria [14]. An interesting observation in this work was that the presence of this bioturbator had no effect on oil removal from the microcosms. In another study, however, the clam *Meretrix meretrix* and polychaete *Perineresis aibuhitensis* have been shown to lead to perturbations of sediment biogeochemistry but did not significantly change microbial communities [43]. It seems that changes in microbial communities in bioturbated sediments depend on the bioturbator species: *H. diversicolor* tends to change the microbial composition in microcosms, whereas *L. louisianensis* does not.

**Figure 4:**
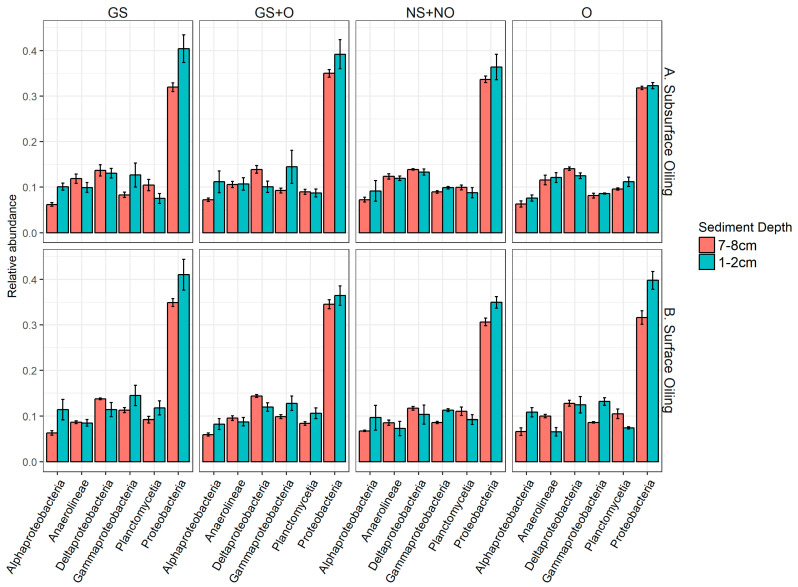
Relative abundances (means ± S.E.) of the most abundant phyla collected from two different depths during experiments with subsurface (**A**) and surface oiling (**B**). Samples were collected from four treatments: “GS”, “GS + O”, “NS + NO”, and “O”, representing “shrimp +, oil −”, “shrimp +, oil +”, “shrimp −, oil −”, and “shrimp −, oil +”, respectively. Only taxa that represent more than 2% of total community are shown.

**Figure 5:**
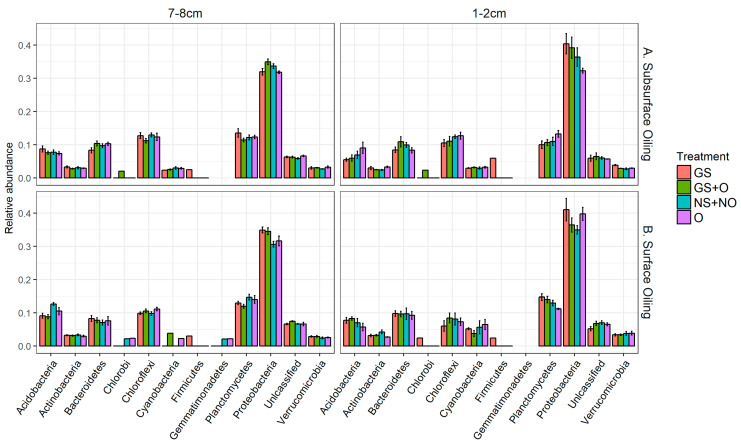
Relative abundances (means ± S.E.) of the most abundant taxa in two greenhouse experiments in which oil was added to the subsurface (**A**) and surface (**B**). For both experiments, sediment samples were collected from four treatments: “GS”, “GS + O”, “NS + NO”, and “O”, representing “shrimp +, oil −”, “shrimp +, oil +”, “shrimp −, oil −”, and “shrimp −, oil +”, respectively, at two separate depths of “1–2 cm” and “7–8 cm”.

Since there were no major differences between bacterial communities in different treatments (Figure 3 and Figure 4), more detailed investigations were carried out to identify small but statistically significant changes in the most abundant individual taxa. Proteobacteria were the most abundant phylum present in all samples, irrespective of bioturbation or oil addition. Other abundant phyla were Planctomycetes, Chloroflexi, Bacteroidetes, Acidobacteria, Actinobacteria, Cyanobacteria, and Verrucomicrobia in descending order of relative abundance (Figure 4). There were differences in the community composition between the surface (1–2 cm) and subsurface samples (7–8 cm; Figure 5). Proteobacteria tend to be more abundant at the surface, primarily driven by high numbers of Alphaproteobacteria. Another group of proteobacteria, Deltaproteobacteria, was more abundant in the subsurface libraries. Marine-sediment Deltaproteobacteria are mostly anaerobic sulfate reducers, which likely explains their tendency to be more dominant in subsurface sediments. The abundance of proteobacteria appears to be increased in the presence of ghost shrimp and with exposure to oil at both surface and subsurface depths, but this cannot be confirmed statistically.

A shift in the microbial community is one of the first environmental responses to an accidental oil spill [41]. This change in the microbial community is a response to selection favoring hydrocarbon-degrading microbes. Immediately after the DWH spill, microbial communities in the water column were driven by the presence of hydrocarbons and nitrogen [44]. There was a distinct succession in the microbial community in the water column, starting shortly after the accident and lasting until most of the spilled oil was depleted [45,46]. Microbial populations also changed in the coastal sediment in response to the DWH oil that settled there, including a ten-fold increase in abundance of *Alcanivorax* spp. Two other key players, *Marinobacter* and *Rhodobacteraceae*, were also present in post-spill sediment [3]. A study following the Exxon Valdez oil spill reported that *Alcanivorax* spp. was an active hydrocarbon degrader there, and this obligate oil-degrading taxon was isolated and identified throughout the world [15,46]. This taxon is present in very low numbers in unpolluted areas, but it is highly efficient in blooming after oil spills. Oil-degrader *Marinobacter* is highly efficient in degrading a wide range of alkanes (C_9_–C_40_), and can survive in hypersaline environments with up to 20% salinity [47,48]. Another gammaproteobacterial member found in high abundance during the DWH accident was an *Acinetobacter* species, and, compared to other taxa, can utilize a broad range of oil hydrocarbons as a carbon source [3,49,50]. Overall, there was a big surge in Gammaproteobacteria in areas with oil from the spill.

No major shifts in the microbial community composition were observed between the control and oil-dosed mesocosms in the present study (Figure 2 and Figure 3). This finding contrasts with the work of Kostka and colleagues [3], who detected a direct response of microbial community composition to oil contamination in environmental settings, which may likely relate to their samples being collected approximately 3 months after the spill, whereas in our experiment, oil exposure lasted only 8 days. Another contributing factor could also be the quantities of oil in the sediment: these authors report that the collection site was “exposed to heavy oil contamination” [3]. Changes in the microbial community following exposure to oil have also been described in laboratory settings using mesocosms and microcosms [14,18]; however, those studies used crude oil concentrations of 7 to 24 mg per g of wet sediment or higher [14,18], which are substantially higher than the concentrations used in the present study (31–52 ng/g wet sediment). The use of relatively low levels of oil was based on preliminary survival experiments with the ghost shrimp (Figure 3) and the study objectives’ need for oil levels that did not interfere with ghost shrimp survival and activity. Moreover, low oil levels are representative of the (more common) areas that are not heavily oiled, and for conditions several years after a blowout. The present study indicates that under these lightly oiled conditions, community-level selection appears to be too weak for a change in microbial community composition. This may especially be the case if the existing community already has some capacity for hydrocarbon degradation. The latter would be expected for the GoM, where the presence of natural hydrocarbon seeps may result in an elevated baseline level of hydrocarbon-degrading microbes. The present study’s most abundant OTU, regardless of treatment, belonged to *Anaerolineae*, a class in the phylum Chloroflexi. A sulfate-reducing anaerobic member of this class appears to be associated with diesel-contaminated soils [51]. The next major OTU present under all treatments belonged to the genus *Synechococcus*, although cyanobacteria as a whole represented only a minor fraction of all OTUs. This genus is widespread in the marine environment, but its abundance tends to decrease dramatically when exposed to oil and high temperatures [51,52]. However, in contrast to the study conducted by Kostka et al. 2011 [3], we did not find any significant rise in *Alcanivorax*, *Marinobacter*, and *Acinetobacter*. These OTUs were present in all our libraries regardless of oil addition or bioturbation, but only in very low numbers (≤48, ≤38, and ≤40, respectively).

The present study also did not detect any major differences in microbial community composition between sediments from mesocosms with ghost shrimp and those without them. Earlier studies have demonstrated that the presence of bioturbators often results in a change in microbial community composition [12,23,24], while in other situations, their presence does not have this effect [43]. It has been well-established that ghost shrimp bioturbation has a substantial impact on sediment physicochemical variables [53,54]. The sediment used in the present study’s mesocosms came from an area with a dense population of ghost shrimp. We hypothesize that the microbial community was slow to respond once ghost shrimp were no longer present in this sediment. Further research on the effects of ghost shrimp on the sediment’s microbial community is ongoing.

In conclusion, while bioturbation and oil addition did not lead to a significant shift in microbial community structure, there were changes in the abundance of a few individual bacteria. The radiotracer assay clearly showed that bioturbation by ghost shrimp enhanced the removal of naphthalene in previously oiled sediments, suggesting that oil exposure, along with bioturbation, stimulated the expression of genes responsible for naphthalene degradation rather than significant changes in the microbial community composition. Further research is needed to identify these genes. One potential candidate is the multicomponent aromatic ring-hydroxylating dioxygenase (RHD). This enzyme is responsible for the primary step of catabolizing PAH degradation—the incorporation of molecular oxygen into the aromatic nucleus. Since this enzyme has a broad spectrum, an increase in the activity of RHD by bioturbation would also be expected to enhance the removal of PAHs that are more complex than naphthalene. The present study demonstrated that the ghost shrimp *L. louisianensis* influenced the fate of crude oil in beach sediment. This Gulf shore bioturbator enhanced degradation of the PAH naphthalene in oiled sediment. Ghost shrimp maintain robust burrows, actively ventilate these with water, and turn over and mix large amounts of sediment. This leads to not only the transfer of hydrocarbons and oxygen into deeper layers, but also the redistribution of associated microorganisms. This study demonstrates the importance of bioturbating organisms with respect to the bioremediation of toxic oil components like naphthalene. We are currently conducting a detailed study of microbial community transcriptomic profiles to confirm the induction of microbial genes involved in petroleum-product removal. Another aspect of our ongoing project is addressing changes in the microbial community composition in response to ghost shrimp bioturbation directly in the natural environment, and its consequences with respect to oil biodegradation.

## Data Availability

The original data presented in the study are openly available in NCBI at PRJNA884405 and SUB16007238.

## Supplementary Materials

The following supporting information can be downloaded at: https://www.mdpi.com/article/10.3390/microorganisms14030695/s1, Table S1: Tukey HSD/Tukey Kramer.
